# Advances in Genetic Risk Scores for Alzheimer’s Disease and Dementia: A Systematic Review

**DOI:** 10.3390/neurolint17070099

**Published:** 2025-06-26

**Authors:** Stefanos N. Sampatakakis, Niki Mourtzi, Alex Hatzimanolis, Nikolaos Scarmeas

**Affiliations:** 1 1st Department of Neurology, Aiginition Hospital, Athens Medical School, National and Kapodistrian University, 11528 Athens, Greece; stefanos.sab@gmail.com (S.N.S.); nikimourtzi23@gmail.com (N.M.); 2 Department of Psychiatry, Aiginition Hospital, Athens Medical School, National and Kapodistrian University, 11528 Athens, Greece; alhatzi@gmail.com; 3 Department of Neurology, The Gertrude H. Sergievsky Center, Taub Institute for Research in Alzheimer’s Disease and the Aging Brain, Columbia University, New York, NY 10027, USA

**Keywords:** genetic risk scores, dementia, Alzheimer’s disease, biological pathways, polygenic risk

## Abstract

Background: Research concerning the genetic risk for dementia has recently been headed towards new directions. Novel findings from genome-wide association studies have highlighted the association of Alzheimer’s disease incidence with many gene polymorphisms, apart from the Apolipoprotein-E genotype. The identification of additional genetic risk factors has led to the construction of specific genetic risk scores for dementia, considering many different genetic factors and specific biological pathways related to Alzheimer’s disease. Methods: We conducted a systematic review following the Preferred Reporting Items for Systematic Reviews and Meta-Analysis method, summarizing existing data regarding genetic risk scores for Alzheimer’s disease and dementia, in order to improve the current understanding of the genetic underpinnings of dementia. In specific, five databases (PubMed/MEDLINE, Embase, Scopus, Web of science, and Cochrane Central) were searched using the keywords “genetic risk score”, “Alzheimer’s disease”, and “dementia” with specific inclusion and exclusion criteria. Results: From the 552 articles identified, we finally included 20 studies for the qualitative analysis. These reports were classified in three different categories of genetic scores: “polygenic risk scores (PRSs)” (including 11 studies), “pathway specific polygenic risk scores (p-PRSs)” (5 studies), and “complex genetic risk scores” (4 studies). Conclusions: Existing genetic risk scores have contributed to better dementia prediction and a better understanding of the underlying pathology. Novel approaches integrating multiple polygenic risk scores might ameliorate the accuracy of genetic risk scores. The combination of polygenic risk scores that are specific to related biological pathways or relevant biomarkers is of utmost importance to achieve a better predictive ability.

## 1. Introduction

The vast majority of dementia cases around the world are attributed to Alzheimer’s disease (AD), which therefore constitutes the most usual cause of dementia [1]. AD is a complex neurodegenerative process that is characterized by gradual cognitive decline [2]. Consequently, AD is of particular interest in terms of public health. This is the main reason why research efforts have intensified during the last decade in order to clarify the underlying etiology and pathophysiology of AD-type dementia [3]. Existing data suggest that genetic factors along with environmental and lifestyle factors all contribute to the clinical manifestation of AD-type dementia [4].

As far as genetic risk for AD is concerned, it appears that the heritability of late-onset AD is around 74% (ranging from 60 to 80%) [5]. It has been shown that the ε4 allele of the apolipoprotein E (APOE) gene is the predominant genetic risk factor related to late-onset AD [6]. However, this specific gene accounts only for a proportion of the total genetic risk [7]. Many more genetic associations, beyond APOE, contribute to AD and, consequently, dementia pathology, as recent genome-wide association studies (GWASs) [8,9,10] have identified at least 40 single-nucleotide polymorphisms (SNPs) associated with AD, instilling a better understanding of how genetics are associated with AD pathology.

Therefore, AD has been recognized as a polygenic disease, and SNPs associated with an increased risk of developing AD-type dementia have been used for the construction of polygenic risk scores (PRSs) [11]. In particular, a PRS is constructed by summing multiple common genetic polymorphisms related to a specific phenotype, weighted by their effect size from independent GWASs [12]. To date, PRSs have appeared to strengthen AD diagnostic classification beyond the use of APOE, as PRSs can discriminate AD cases from controls, with a prediction accuracy of approximately 70–80% [13]. Apart from this, PRSs have also been associated with longitudinal cognitive decline [14], as well as specific biomarkers of AD, such as cerebrospinal fluid (CSF) amyloid-beta (Aβ) and Tau, and markers from neuroimaging studies [15,16].

In the existing literature, PRSs have also been used in the context of genetic risk scores (GRSs) [17]. A GRS is a way of summing the genetic risk that an individual carries for a particular disease [18] by combining relatively small effects of different genetic factors (genes or loci) through composite consideration. Under this perception, those factors, when considered collectively, could account for substantial differences in disease risk. Thus, GRS is an overarching term, which includes the PRS approach.

Undoubtedly, existing studies [13,14,15,16,17] have provided an essential contribution to the literature for AD genetic risk. The association of PRSs with AD incidence as well as relevant biomarkers has already been shown. Moreover, the assumption that risk stratification for AD might be improved by combining genetic factors with additional data (such as biomarkers or lifestyle factors) has also been made. The sole relevant systematic review summarizing data regarding the genetic risk for AD beyond APOE ε4 was published in 2018 [19]. It included 18 genetic studies (from 2010 to 2018) in adult populations of European descent with detailed genotyping techniques and a longitudinal design. The main conclusion was that all GRSs consisting of SNPs associated with AD through relevant GWASs expressed significant discrimination capability for AD incidence, in relation to cognitively normal status; however it was also stated that further validation of the results in population-based longitudinal studies was needed.

The previously conducted systematic review also presents notable limitations. The absence of a common method for the selection of included SNPs (apart from the criterion of statistically significant association with relevant GWASs) might render the choice of specific thresholds, in part, inconsistent. Moreover, the variance in statistical measures of reported associations makes the results difficult to compare. Concerns regarding generalizability may also arise, as study populations included mostly individuals of European descent, and were often recruited from clinical settings (such as memory clinics).

Furthermore, novel approaches for genetic risk calculation were not included. For instance, a PRS might include a mixture of SNPs acting in different or even opposite directions at a biological level, without incorporating biological information. Therefore, recently, a methodology has been developed in order to group the SNPs into different biological processes, such as amyloid aggregation, inflammation, and metabolism [20], and construct “pathway-specific” PRSs (p-PRSs) that disentangle the action of different biological pathways [21]. At the same time, since 2019, machine learning methods have been used in order to combine multiple PRSs in one genetic score [22,23].

Thus, we conducted a particular systematic literature review in order to enrich existing knowledge concerning genetic scores for dementia. Our aim was to include the newer methods for genetic risk calculation, as well as to cope with the aforementioned limitations of the existing literature. Given that an update to the systematic review of 2018 has not been published, we summarized the findings of original research studies that have investigated a GRS for AD or all-type dementia since mid-2018 (2018–2024).

## 2. Materials and Methods

### 2.1. Systematic Review Search Strategy

The specific systematic review was performed based on the Preferred Reporting Items for Systematic Review and Meta-analysis Protocols (PRISMA) statement [24]. The PRISMA 2020 checklist can be found in Table S3 of the Supplementary Materials and the PRISMA 2020 checklist for abstracts is presented in Table S4. The protocol of the systematic review was registered in PROSPERO (ID: CRD420251005424).

**Figure 1:**
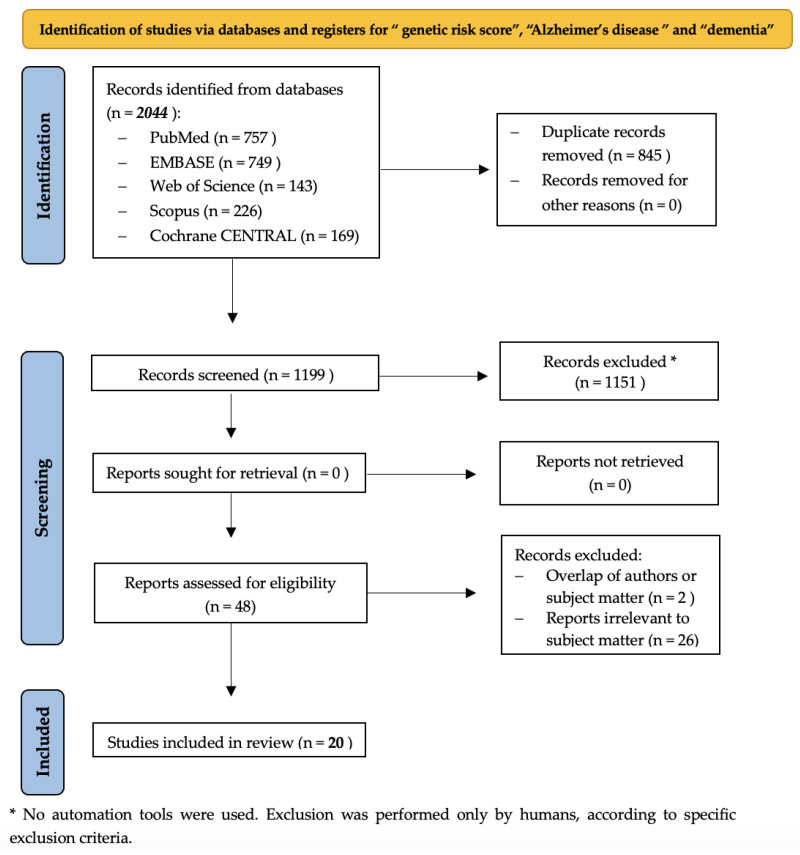
PRISMA flow diagram of systematic review.

In the phase of identification, five electronic bibliographic databases, PubMed/MEDLINE, EMBASE, Scopus, Web of Science, and Cochrane Central, were searched from each database inception until 1 March 2025 (the exact dates can be found in Section S1 of the Supplementary Materials). The search term “genetic risk score” was used in combination with the terms “Alzheimer’s disease” and “dementia”. Overall, 2044 records were identified. The exact search strings can be found in the Supplementary Materials, while the different phases of the systematic review performed are described in Figure 1.

### 2.2. Screening and Eligibility Strategy

The phase of screening and eligibility was blindly conducted by two individual researchers (S.N.S. and N.M.). To promote reliability, the researchers examined all titles and abstracts retrieved from the search on the same day.

Overall, 845 duplicate records were removed. From the remaining 1199 reports, the full text was available in 1173 articles. Then, we selected publications only since 7 April 2018 (*n* = 552), given that the previously conducted systematic review included relevant studies until 6 April 2018. At that point, we applied the inclusion and exclusion criteria presented in Table S1 of the Supplementary Materials. In total, 504 records were excluded, leading to 48 full-text articles identified as relevant.

The 48 articles from the first screening stage were further assessed for eligibility. In specific, studies were evaluated for potential overlap of authors or subject matter, as well as relevance to the subject matter, leading to the exclusion of 28 more reports (for example concerning early-onset AD or investigating different outcomes). As this specific review examines the relationship between GRSs, AD, and dementia, articles examining only one genetic risk factor (such as the APOE ε4 allele) were excluded as irrelevant to the subject matter. In case of disagreement during the eligibility assessment, another investigator (N.S.) reviewed the full text in question and gave final approval. Finally, 20 studies were included in qualitative analysis.

### 2.3. Risk of Bias Assessment

The levels of bias in the included studies were assessed using the study limitation concerns presented in the Newcastle–Ottawa Scale (NOS) [25], as well as the Q-Genie tool [26] for genetic studies. The risk of bias assessment was performed by two independent researchers (S.N.S. and N.M.). Bias items were estimated separately for each included study.

The NOS consists of three sub-sections: selection (including four items), comparability (including two items), and exposure (comprising three items), which are slightly different according to the type of study (case–control, cohort, or cross-sectional study). Each item was rated with one star, reflecting the methodological quality of the study (total points 0–9 or 0–10). At the same time, the Q-Genie tool consists of 11 distinct questions assessing the quality of genetic studies. According to the Q-Genie assessment, good quality is determined by a score higher than 40 for genetic studies without control groups and 45 for studies with control groups, respectively. The detailed risk of bias assessment for all included studies is presented in Tables S2 and S3.

## 3. Results

In total, 20 studies were selected for the final stage in order to achieve the objectives of this systematic review. The results were stratified into three subsections of genetic scores: (i) “PRSs”, including scores derived from relevant GWAS-significant SNPs, (ii) “p-PRSs”, including scores associated with specific pathways that are thought to be implicated in AD, and (iii) “complex GRSs”, including scores combining multiple genetic factors, as well as scores that cannot be classified in either of the other two categories.

### 3.1. PRSs

In total, 11 studies regarding specific PRSs for AD derived from a GWAS were included [17,27,28,29,30,31,32,33,34,35,36]. The main outcomes investigated were as follows: (a) cognitive outcomes (AD or all-type dementia risk and cognitive function) and (b) biomarker outcomes (CSF or neuroimaging). The most widely used GWAS for GRS construction was conducted by Kunkle et al. [9] as part of the 2019 International Genomics of Alzheimer’s Project (IGAP) concerning late-onset AD in Europeans, followed by the previous version of IGAP conducted by Lambert et al. [8] in 2013. Apart from the above-mentioned GWASs, data from six additional GWASs [37,38,39,40,41,42] were used for GRS construction in the included studies.

#### 3.1.1. Studies Investigating Cognitive Outcomes

To begin with, eight studies [17,27,28,29,30,31,32,33,34,35,36] aimed to associate genetic scores including or excluding the APOE genotype with cognitive outcomes, including AD or dementia risk as well as cognitive performance, as shown in Table 1.

In particular, three GRSs derived from three different GWASs including both European and American participants were associated with all-dementia risk, either as a continuous variable [17,27] or as quintiles [28]. At the same time, AD risk was also associated with GRSs for AD in European and Asian populations [29,30,31], either independently or in combination with dietary factors.

Regarding specific metrics of cognitive function, the two included studies used cross-sectional designs. A GRS consisting of 23 SNPs was associated with the episodic memory and dementia-related variability in cognitive function of an Australian population [32], while another GRS was related only to worse Montreal Cognitive Assessment (MoCA) but not composite cognitive score in middle-aged participants from San Francisco [33].

#### 3.1.2. Studies Investigating Biomarkers as Outcome

AD biomarkers have been investigated in the form of CSF, plasma, or neuroimaging markers [from Magnetic Resonance Imaging (MRI) or Positron Emission Tomography (PET)]. Relevant studies are shown in Table 2. Concerning CSF biomarkers, CSF P-Tau_181_ was positively related while CSF Aβ_42_ and the ratio of CSF Aβ_42_/Aβ_40_ were inversely related to a GRS consisting of 20 SNPs in a study using data from Chinese participants over 60 years old [31].

Plasma P-Tau_181_ was associated with a GRS consisting of 21 SNPs based on the GWAS of De Rojas et al. [40] in participants from the Mayo Clinic Study of Aging [34]. Greater amyloid PET levels were also related to the GRS. At the same time, high GRS in combination with high plasma P-Tau_181_ improved the classification accuracy of amyloid PET positivity in comparison to plasma P-Tau_181_ alone.

On the contrary, an AD-GRS based on the GWAS conducted by Bellenguez et al. [41] was not related to amyloid PET deposition or MRI volumes [35] (cortical, total gray matter, and hippocampal) in a Finnish population without dementia at baseline. However, a GRS constructed based on the GWAS of Kunkle et al. [9] was associated with greater age-related reduction in specific brain MRI volumes (medial orbitofrontal cortex, hippocampus, nucleus accumbens, thalamus, and amygdala) in UK Biobank participants with valid MRI data [36].

### 3.2. P-PRSs

In total, five studies concerning p-PRSs for AD were included [20,21,42,43,44] in our review, as shown in Table 3. The most commonly used GWAS was the one conducted by Kunkle et al. [9]. The main method used for the construction of p-PRSs was variant–pathway mapping, in which SNPs were firstly associated with genes (variant–gene mapping) and then genes were associated with pathways (gene–pathway mapping) [20]. The results of included studies are classified based on the outcome investigated. The main outcomes were either cognitive (AD diagnosis, AD risk, or cognitive changes) or biomarkers.

In specific, AD diagnosis was investigated in the study conducted by Schork et al. [42] and it was cross-sectionally related to 8 of the 13 p-PRSs calculated. AD risk was explored in the study of Tesi et al. [20] and was associated with all five p-PRS constructed. Among these, only angiogenesis was not related to AD risk when excluding APOE.

Concerning cognitive function, in the study of Xu et al. [21] including six p-PRS, it was shown that three different pathways (endocytosis, Aβ_PP_ metabolism, and Tau pathology) were related to changes in specific domains (immediate learning, delayed recall, and executive function), as presented in Table 3. Moreover, higher values of p-PRSs were associated with an earlier effect of age on cognitive function. In the study of Sun et al. [43], a p-PRS for Tau-protein binding and kinase activity was associated with decline in the memory domain.

Regarding biomarkers of AD, these were explored in four studies. In the study of Schork et al. [42], 13 p-PRSs were included, among which 4 (endocytosis, misfolded protein, regulation protein tyrosine, and fibril formation) were related to positivity in either CSF or PET amyloid, while 3 were related to P-Tau positivity and 2 p-PRSs were associated with lower hippocampal volume. Furthermore, in the study of Sun et al. [43] a p-PRS specific to Τau-protein kinase activity and Tau-protein binding was related to baseline CSF and PET Tau as well as a greater longitudinal increase in CSF Tau. At the same time, cortical brain atrophy was also associated with specific p-PRSs (for cholesterol and Aβ_PP_ metabolism) in the study of Caspers et al. [44], particularly regions between the inferior frontal and adjacent precentral sulcus and the posterior temporal and medial occipital cortex. Last but not least, four p-PRSs investigated by Xu et al. [20] were related to age-dependent changes in CSF biomarkers (CSF Aβ_42_ and Aβ_42_/_40_, Tau, and P-Τau).

### 3.3. Complex GRSs

In this specific subsection we included four studies [22,23,46,47], which are presented in Table 4. To begin with, a weighted risk score for APOE (the APOE-npscore) was constructed using data from a study of APOE genetic risk in autopsy AD cases [48]. The odds ratio (OR) values reported using the ε3-ε3 genotype as reference were log (ln)-transformed [46]. The specific score was able to explain more variance of CSF P-Tau_181_ and the CSF ratios of Aβ_42_/_40_ and P-Tau_181_/Aβ_42_ in relation to the status of APOE ε4 carriership or the ε4 allele count.

Another complex score was constructed by Zhang et al. [47] using a novel approach. In particular, the investigators selected seven SNPs from many GWASs using specific inclusion criteria (such as genome-wide significance in both European and Asian population) and constructed a weighted GRS (wGRS). In the wGRS calculation, the effect size from logistic analysis was multiplied with the average number of risk alleles (0, 1, or 2) in each selected SNP. The difference between the wGRS method and the traditional PRS method lies in the fact that when calculating a PRS, the effect size from relevant GWASs is multiplied with either 0 (in the absence) or 1 (in the presence of the specific SNP) in each individual. Interestingly, the area under curve (AUC) for wGRS was significantly greater in comparison to the AUC of GRS calculated based on the traditional PRS method.

Concerning the combination of multiple PRSs in the same GRS, to date, the specific approach has been investigated in two studies. A meta-GRS for AD was calculated using machine learning methods (elastic-net logistic regression followed by k-fold cross-validation), incorporating 25 distinct PRSs specific for risk factors and comorbidities correlated with AD [22]. The meta-GRS had similar predictive power to the AD-GRS alone, which was based on the GWAS of Kunkle et al. [9] and outperformed the predictive power of a reference model without genetic factors.

Recently, D’Aoust et al. [23] also used machine learning in order to combine PRSs for 27 dementia-related traits in one score (iPRS-DEM), capturing both vascular and neurodegenerative aspects (such as AD, hippocampal volume, CSF P-Tau, and CSF Aβ). The iPRS-DEM score was associated with dementia risk (independently of APOE) in older community-dwelling people (HR: 1.15), a result which was validated in two cohorts (with improved performance in dementia-free memory clinic participants). When combined with APOE ε4 positivity, the iPRS-DEM resulted in a five-fold increased dementia risk in memory-clinic participants.

## 4. Discussion

In our systematic review, we sought to summarize existing evidence on genetic risk scores for AD and dementia, providing an update to the previous relevant systematic review published in 2018 [19]. In comparison to the conclusions of the 2018 review, we also found that PRSs have been consistently associated with AD and dementia risk, extending previous findings to non-European populations. Moreover, we aimed to enrich the existing literature by summing up available data regarding the relationship of PRSs with cognitive decline and AD biomarkers. At the same time, we also added new methods investigating genetic risk for dementia such as p-PRSs and complex GRSs (consisting of many PRSs and additional genetic factors), which seem really promising and might be useful in explaining a higher proportion of dementia variability as well as the underlying pathophysiology. However, their clinical utility remains unvalidated.

### 4.1. GRSs in the Context of PRSs

The importance of PRSs in disease prediction has been well established in past research [12,13]. The effect of PRSs in disease risk is independent of the individual’s age and the time of assessment, providing better large-scale applicability. Moreover, PRSs might add to genetic risk stratification beyond APOE ε4, as the combination of PRSs and APOE genotype have reached greater AUCs in several studies [31,47] in comparison to the predictive power of APOE genotype or PRS alone.

This is the main reason why PRSs have become increasingly important in quantifying AD or dementia risk, especially in people of European origin, given that the vast majority of existing GWASs are based on European participants. In fact, around 80% of conducted GWASs are restricted to European participants despite the fact that only 11.5% of the world population is of European origin [19], which is a major concern concerning PRS analysis. Recently, research efforts have been made to obtain data from Asian, Australian, and American populations, with the first results already published and included in our review [9,39].

Apart from disease risk, PRSs have also been investigated in regard to environmental and lifestyle risk factors, such as body mass index (BMI) [49], sleep duration [50], cardiovascular health [27,28], and dietary factors [29,30], which also play a significant role in disease progression. Although, to date, no GRS has shown interaction with the above-mentioned lifestyle factors, a modifying relationship has been established. In specific, modifiable lifestyle risk factors, such as adherence to the Mediterranean diet, have appeared to modify the effect of genetic risk on AD incidence and may also have direct effects on vascular cognitive impairment and brain aging [28]. Vice versa, the effect of modifiable lifestyle risk factors on cognition might be modified by increased genetic risk, as well. For example, the association of the Mediterranean diet with AD risk was more prominent in adults with a low polygenic risk for AD in the HELIAD study [30]. Therefore, genetic risk for AD should be considered as an important factor that may affect the outcome when planning interventions targeting modifiable risk factors for dementia in order to improve cognition.

In any case, PRSs for AD have not been able to investigate the full landscape and underlying pathophysiology of dementia. As stated in the systematic review of 2018 [19], an explanation might be that several informative genetic factors may fall beneath the genome-wide significance thresholds. However, an approach not restricted to SNPs reaching genome-wide significance might lead to misleading results due to the inclusion of many non-informative SNPs, as shown in the study of Saadman et al. [35] using a more liberal approach.

Lately, research efforts in AD have shifted towards a biological definition, as the recently published revised criteria for diagnosis and staging of AD [51] from the National Institute on Aging and Alzheimer’s Association Research Framework have focused on the use of biological parameters, such as biomarkers. Under this perception, PRSs related to specific pathways implicated in AD have been developed.

### 4.2. P-PRS for AD-Related Pathways

Treatments targeting amyloid have, so far, not been able to slow or stop disease progression. Thus, the inability of the amyloid cascade hypothesis to explain AD pathology has increased the interest in other pathways that might be important in AD pathogenesis. In the direction of better understanding AD etiology, p-PRSs have been constructed, with relevant studies showing that apart from amyloid metabolism, additional pathways (i.e., immune response and endocytosis) significantly contribute to AD genetic risk [20,42].

To date, p-PRSs have been associated with AD risk [20,41], as well as cognitive outcomes [21]. Additionally, p-PRSs have been related to specific AD biomarkers, such as CSF or PET Aβ, P-Tau [43], and Tau [44], as well as brain volumes in neuroimaging [33,44], but not consistently. Interestingly, in the study of Xu et al. [21] the effect of two p-PRSs (for Aβ_PP_ and cholesterol metabolism) on CSF amyloid appeared to be similar to that of APOE, but with a later onset (approximately 5–10 years later).

The p-PRS method may provide useful information beyond variants arising from GWASs. However, several concerns regarding the use of p-PRS have been identified, as p-PRSs have not yet appeared to add any predictive power in relation to PRSs for AD [43]. Moreover, even though in existing studies the vast majority of SNPs have been shown to be associated with only one biological pathway, some SNPs are part of multiple pathway clusters. Consequently, p-PRSs are not entirely independent of each other; therefore, an overlap between the contribution of different p-PRSs is possible.

### 4.3. Other Complex GRSs

Given that the aforementioned methods have not provided undisputed results, the idea of combining many different genetic factors in one complex genetic score has emerged in an effort to increase the predictive power of GRSs for AD and dementia. The combination of multiple PRSs in one GRS requires advanced statistical analyses, including machine learning models.

This novel approach is still in its infancy, as only two relevant studies have been published. Firstly, a meta-GRS for AD was constructed including 25 distinct PRSs for risk factors and comorbidities correlated with AD [22]. The meta-GRS did not show superior predictive power in comparison to the AD PRS itself [22]. Secondly, the iPRS-DEM was constructed based on 27 dementia-related traits and was also associated with increased dementia risk, although the hazard ratio was relatively small [23]. At the same time, the iPRS-DEM showed reduced predictive power than a model including clinical factors associated with increased dementia risk; however, it slightly improved the AUC when added to a model comprising only clinical factors (AUC: 0.756, in comparison to 0.751 of the clinical model).

Thus, conducted studies have not yielded impressive results regarding the variability explained. Nevertheless, the combination of many PRSs might pave the way for more accurate genetic scores. For instance, the methodology introduced can be utilized in future studies combining PRSs that are specific to AD pathways or biomarkers, in order to elucidate the pathogenesis of AD and dementia to a greater extent. Thus, the selection of PRSs is of utmost importance to achieve better predictive ability, since the aforementioned studies [22,23] included PRSs that were not closely related to AD or dementia.

### 4.4. Limitations and Strengths of Our Study

This systematic review is not without limitations. Included studies were heterogeneous in many ways. The main sources of heterogeneity were the following: (a) in the statistical analyses, as in the conventional PRS method, regression models were used (logistic or proportional hazard models), in comparison to more advanced methods and machine learning models used for complex GRSs; (b) many different outcomes were investigated, such as AD diagnosis or risk, cognitive status (global or domain-specific), and associations with distinct AD biomarkers; and (c) the study populations were diverse, as in several studies the sample was population-based, while in other studies the population was derived from a clinical setting (memory outpatient clinics), leading to possible selection bias. Thus, the heterogeneity of included studies might affect the results, causing misleading conclusions.

Nevertheless, our systematic review search strategy followed the PRISMA guidelines, which are well-established and minimize the risk of bias. Additionally, extensive assessment of the risk of bias was performed using a well-established scale, which makes us confident regarding the quality of results. Concerning the additional strengths of our review, all included studies used comprehensive techniques for genotyping. Moreover, we presented results derived from various populations, including participants of European, American, Asian, and Australian ancestry, enhancing existing knowledge, which was limited to individuals of European origin. Last but not least, to our knowledge, this is the first systematic review regarding GRSs for AD and dementia including novel approaches, such as p-PRSs and complex GRSs.

## 5. Conclusions

This particular systematic review focused on summarizing existing knowledge regarding GRSs for AD and dementia, providing an update to the previous systematic review of 2018. The main addition of our review was the integration of novel approaches for genetic risk estimation, such as p-PRS and complex GRSs. Complex GRSs incorporating multiple PRSs appear to be really promising, enhancing the existing potential for GRS use in a clinical setting as a genetic risk stratification tool in order to identify individuals with the highest susceptibility, and to potentially intervene earlier to prevent cognitive decline. In any case, the complex GRS method is subject to improvement, and additional population-based prospective studies are needed to validate its clinical utility.

## Figures and Tables

**Table 1:** Studies investigating PRSs with cognitive outcomes.

Study	Population Characteristics	GWASs ^1^	SNPs ^2^	Result for GRS ^3^ by outcome
Mukadam et al., 2022 [17]	*N* = 364.879 From UK Biobank	Lambert et al. [8]	21 SNPs (*p* < 5 × 10^−8^)	GRS related to increased dementia risk (OR ^4^: 1.21 for each SD ^5^ increase)
Tin et al., 2022 [27]	*Ν* = 11.561 8.823 European Americans (EA) 2.738 African Americans (AA)	Kunkle et al. [9]	44 SNPs for EA 9 SNPs for AA	GRS associated with higher dementia risk (EA, HR ^6^: 1.44 for each SD increase; AA, HR: 1.26 for each SD increase)
Peloso et al., 2020 [28]	*N* = 1.211 From Framingham Heart Study Over 60 years old	Van der Lee et al. [37]	23 SNPs	High GRS (>80th percentile) associated with a 2.6-fold-higher dementia risk compared to the lowest quantile (<20th)
Peng et al., 2023 [29]	*N* = 207.301 From UK Biobank 39–72 years old	Jansen et al. [10]	29 SNPs	Higher GRS with higher dietary inflammatory indexes were related to a higher AD ^7^ risk (HR: 1.757)
Mamalaki et al., 2023 [30]	*N* = 537 From HELIAD study Over 65 years old	Wightman et al. [38]	38 SNPs (*p* < 0.05)	GRS associated with increased AD risk (HR: 1.928 for each SD increase)
Li et al., 2024 [31]	*N* = 3020 From China Over 60 years old	Chinese GWAS [39]	20 SNPs (*p* < 5 × 10^−8^)	High GRS related to increased late-onset AD risk (OR: 3.15)
Andrews et al., 2019 [32]	*N* = 1061 From Australia Community-dwelling, >60 years	Lambert et al. [8]	23 SNPs 2 for APOE	GRS cross-sectionally associated with episodic memory (−0.10) and cognitive variability (−0.08)
Brenowitz et al., 2023 [33]	*N* = 1252 From San Francisco Middle-aged	Kunkle et al. [9]	25 SNPs	GRS related cross-sectionally to worse Montreal Cognitive Assessment (−0.14 SD), but not composite cognitive score

^1^ Genome-Wide Association Studies, ^2^ Single-Nucleotide Polymorphisms, ^3^ Genetic Risk Score, ^4^ Odds Ratio, ^5^ Standard Deviation, ^6^ Hazard Ratio, ^7^ Alzheimer’s Disease.

**Table 2:** Studies investigating PRSs with biomarkers as the main outcome.

Study	Population Characteristics	GWASs ^1^	SNPs ^2^	Result for GRS ^3^ by outcome
Li et al., 2024 [31]	*N* = 3020 From China, over 60 years old	Chinese GWAS [39]	20 SNPs (*p* < 5 × 10^−8^)	GRS related to CSF ^4^ P-Tau_181_ and inversely related to CSF Aβ_42_, CSF Aβ_42_/Aβ_40_ ratio
Ramanan et al., 2023 [34]	*N* = 962 From Mayo Clinic Study of Aging	De Rojas et al. [40]	21 SNPs (*p* < 5 × 10^−8^)	GRS related to greater amyloid PET ^5^ levels and plasma P-Tau_181_
Saadman et al., 2024 [35]	*N* = 1260 From Finland	Bellenguez et al. [41]	83 SNPs	GRS not related to PET amyloid deposition or MRI ^6^ volumes
Buto et al., 2023 [36]	*N* = 47.502 From UK Biobank	Kunkle et al. [9]	26 SNPs (2 for APOE ^7^)	GRS associated with age-related reduction in specific MRI regions

^1^ Genome-wide association studies, ^2^ single-nucleotide polymorphisms, ^3^ genetic risk score, ^4^ cerebrospinal fluid, ^5^ Positron Emission Tomography, ^6^ Magnetic Resonance Imaging, ^7^ apolipoprotein E.

**Table 3:** Studies investigating p-PRSs.

Study	Population Characteristics	GWASs ^1^	p-PRS ^2^	Cognitive Outcome	Biomarker Outcome
Tesi et al., 2020 [20]	*N* = 1779 From the Longitudinal Aging Study Amsterdam	Many GWAS 29 SNPs ^3^	(1) B-amyloid metabolism (2) Immune response (3) Cholesterol dysfunction (4) Endocytosis (5) Angiogenesis	All p-PRSs were related to increased AD ^4^ risk	
Xu et al., 2023 [21]	*N* = 1175 From the Wisconsin Registry for Alzheimer’s Prevention	Kunkle et al. [9] 23 SNPs	(1) Aβ_PP_ metabolism (2) Immune response (3) Cholesterol metabolism (4) Endocytosis (5) Tau pathology (6) Axonal development	P-PRS related to preclinical cognitive changes in - Immediate learning [Endocytosis] - Delayed recall [Aβ_PP_, Tau, endocytosis] - Executive function [Endocytosis, Tau]	P-PRSs related to age-dependent changes in - CSF ^5^ Aβ_42_ and Aβ_42_/_40_ [Aβ_PP_, immune response, cholesterol metabolism] - CSF ^5^ Tau and P-Τau [Aβ_PP_, immune response, endocytosis]
Schork et al., 2023 [42]	*N* = 1411 From the Alzheimer’s Disease Neuroimaging Initiative (ADNI) European ancestry	Kunkle et al. [9] 351.203 SNPs	(1) Amyloid processing (2) Inflammatory response (3) Protein localization (4) Cholesterol transport (5) Immune signaling (6) Endocytosis (7) Humoral immune response (8) Receptor metabolic process (9) Response to misfolded protein (10) Phototransduction (11) Regulation of cell junction (12) Regulation of protein tyrosine (13) Mitophagy	8 p-PRSs related to baseline AD diagnosis	P-PRSs related to the following biomarkers: - CSF 5 or PET 6 amyloid [endocytosis, fibril formation, response to misfolded protein, regulation protein tyrosine] - CSF or PET P-Tau [protein localization, immune signaling, mitophagy] - Hippocampal volume [protein localization, mitophagy]
Sun et al., 2021 [43]	*N* = 567 From the ADNI	Kunkle et al. [9] 60 SNPs	(1) Tau-protein binding and kinase activity	P-PRS related to memory impairment	P-PRS related to - Baseline CSF/PET Tau - Greater longitudinal increase in CSF Tau
Caspers et al., 2020 [44]	*N* = 537 From 1000BRAINS Older adults from Bochum	Kunkle et al. [9] and Sabuncu et al. [45] 20 SNPs *p* < 5 × 10^−8^	(1) Aβ_PP_ metabolism (2) Immune response (3) Cholesterol metabolism (4) Endocytosis (5) MAPT metabolism (6) Axon development		Two p-PRSs (cholesterol and Aβ_PP_ metabolism) related to regional cortical atrophy

^1^ Genome-Wide Association Studies, ^2^ Pathway-Specific PRS, ^3^ Single-Nucleotide Polymorphisms, ^4^ Alzheimer’s Disease, ^5^ Cerebrospinal Fluid, ^6^ Positron Emission Tomography.

**Table 4:** Studies investigating complex GRSs.

Study	Population Characteristics	Genetic Study	SNPs ^1^	Result for GRS ^2^ by Outcome
Deming et al., 2023 [46]	*N* = 1045 From the Wisconsin Registry for AD ^3^ Prevention and Research	Reiman et al. [48]	APOE ^4^-npscore	The APOE-npscore explained more variance of CSF ^5^ Aβ_42_/_40_, CSF P-Tau_181,_ and P-Tau_181_/Aβ_42_ than APOE ε4 carrier status or ε4 allele count
Zhang et al., 2019 [47]	*N* = 1259 From Inner Mongolia (China)	Many GWAS ^7^ and the NHGRI catalog	Weighted (**wGRS**) with 7 SNPs (3 for APOE)	The wGRS was related to increased AD risk The AUC ^8^ for wGRS was significantly greater than the AUC for simple-count GRS
Clark et al., 2023 [22]	*N* = 5869 From the National Alzheimer’s Coordinating Center (NACC)	Many GWASs for 25 traits * related to AD	Meta-GRS	The meta-GRS was related to a 57% increase in the AD risk for each SD ^9^ increase (HR ^10^ = 1.577)
D’ Aoust et al., 2025 [23]	*N* = 3702 From French cities Community-dwelling, ≥65	Many GWASs for 27 traits ** related to dementia	I-PRS Dem	The iPRS-DEM was related to increased dementia risk in the elderly (HR= 1.15), a result validated in two cohorts

^1^ Single-nucleotide polymorphisms, ^2^ genetic risk score, ^3^ Alzheimer’s disease, ^4^ apolipoprotein E, ^5^ cerebrospinal fluid, 6, ^7^ genome-wide association studies, ^8^ area under curve, ^9^ Standard deviation, ^10^ hazard ratio. * AD, vascular risk factors, MRI volumes, psychiatric disorders, education, sleep, stroke; ** AD, vascular risk factors, MRI markers of cerebral small vessel disease, stroke subtypes, hippocampal volume, CSF P-Tau, and Aβ.

## Supplementary Materials

The following supporting information can be downloaded at https://www.mdpi.com/article/10.3390/neurolint17070099/s1, Table S1. Systematic review inclusion and exclusion criteria; Table S2. Quality rating of included studies: modified Newcastle–Ottawa scale; Table S3. Q-Genie assessment for genetic studies; Table S4. PRISMA 2020 systematic review checklist; Table S5. PRISMA 2020 checklist for abstracts. References [17,20,21,22,23,25,26,27,28,29,30,31,32,33,34,35,41,42,43,44,45,46] are also cited in the supplementary materials.
