# Supplementary material for: Advances in Genetic Risk Scores for Alzheimer’s Disease and Dementia: A Systematic Review
Source: Neurol Int. 2025 Jun 26;17(7):99. doi: 10.3390/neurolint17070099 (PMC12298128; doi:10.3390/neurolint17070099)
Supplement: Supplementary file 1 [file neurolint-17-00099-s001.zip › neurolint-3599118-supplementary.pdf]

SYSTEMATIC REVIEW SUPPLEMENTARY

S1. Systematic Review Search Strategy

Preferred Reporting Items for Systematic Review and Meta-analysis Protocols (PRISMA) guidelines for systematic reviews were applied to conduct the systematic review. Five databases (PubMed/MEDLINE, EMBASE, Web of Science, Cochrane Central and Scopus) were searched as follows:

- **PubMed / MEDLINE**

Time of search: Last search on 1st of March 2025
Range of dates explored: From database inception (1996) until 1st of March 2025
Boolean search string: "genetic risk score" **AND** "Alzheimer's dementia"
Actual MeSH term search:
("genetic risk score"[MeSH Terms] OR ("genetic"[All Fields] AND "risk"[All Fields] AND "score"[All Fields]) OR "genetic risk score"[All Fields]) AND ("alzheimer disease"[MeSH Terms] OR ("alzheimer"[All Fields] AND "disease"[All Fields]) OR "alzheimer disease"[All Fields] OR ("alzheimer s"[All Fields] AND "dementia"[All Fields]) OR "alzheimer s dementia"[All Fields])
Results: 757 entries

- **EMBASE**

Exactly the same Boolean search string has been used in the EMBASE database (last search on 1st of March 2025).
Range of dates explored: From database inception (1974) until 1st of March 2025
Results: 749 entries

- **Web of Science**

Range of dates explored: From database inception (1997) until 1st of March 2025
Boolean search string: "genetic risk score" **AND** "Alzheimer's dementia"
Actual term search: TOPIC (genetic risk score OR genetic risk OR score OR genetic OR risk score OR risk OR score) AND TOPIC (Alzheimer disease OR Alzheimer OR disease OR Alzheimer's dementia OR dementia)
Results: 143 entries

- **Cochrane CENTRAL**

Exactly the same Boolean search string has been used in the EMBASE database (last search on 1st of March 2025).
Range of dates explored: From database inception (1996) until 1st of March 2025
Results: 169 entries

- **Scopus**

Exactly the same Boolean search string has been used in the EMBASE database (last search on 1st of March 2025).
Range of dates explored: From database inception (2004) until 1st of March 2025
Results: 226 entries

Table S1. Systematic Review Inclusion and Exclusion Criteria

INCLUSION CRITERIA	EXCLUSION CRITERIA
• Type of Study<br>– Original peer-reviewed research articles (clinical studies, clinical trials, observational studies)	• Article Type<br>– Reviews<br>– Books and documents<br>– Animal studies
• Main Predictor<br>– Presence of a defined genetic risk score for Alzheimer's Disease (AD) or dementia incorporating genetic variants	• Population Type<br>– Populations focusing on participants with another neurological disease
• Date of Publication<br>– From 07 April 2018	• Date of Publication<br>– Before 07 April 2018
• Language: English<br>• Availability of the full text	• Language: other language

S2. Study data extraction and analysis, outcomes investigated

The researchers extracted and analyzed the following data from the included articles:

– Study name
– Type of genetic study
– Sample size & population characteristics
– Methods of statistical analysis
– The Genome-Wide Association Study (GWAS) of reference
– Number of SNPs included in the genetic risk score (GRS)
– Whether APOE was included in the GRS
– Reported limitations
– Results regarding the association between GRS and the outcome of interest.

In particular, the following outcomes were investigated:

(a) Dementia or AD diagnosis
(b) Dementia or AD risk
(c) Cognitive measures (metrics of cognitive function)
(d) Biomarkers of AD, including cerebrospinal fluid (CSF), plasma or Positron Emission Tomography (PET)
(e) Brain (magnetic resonance imaging) MRI volumes

S3. Risk of bias assessment

The levels of bias were assessed using the study limitation concerns presented in the Newcastle-Ottawa Scale (NOS) [25] as well as the Q-Genie [26] assessment for genetic

studies. The NOS for each included study with the different items examined is presented in **Table S2**, while the Q-Genie scores can be found in **Table S3**. In our case, according to the Q-Genie assessment, all included studies have exceeded the threshold to be considered of good quality, with Q-genie scores ranging from 46 to 57.

Table S2. Quality rating of included studies: modified Newcastle-Ottawa scale

	**SELECTION**				**COMPARABILITY**		**EXPOSURE**			
**CASE-CONTROL**	**Case Definition**	**Representativeness**	**Control Selection**	**Control Definition**	**Controls for AD**	**Controls for GRS**	**Genetic Risk**	**Same Method**	**Missing Data Rate**	**Total Score [9]**
Zhang et al. [47]	1	1	0	1	1	0	1	1	0	**6**
**COHORT STUDIES**	**Control Selection**	**Representativeness**	**Exposure**	**No Outcome**	**Controls for AD**	**Controls for GRS**	**Outcome Assessment**	**Follow-Up Length**	**Follow-Up Adequacy**	**Total Score [9]**
Xu et al. [21]	1	1	1	0	1	0	1	1	1	**7**
Tin et al. [27]	1	1	1	0	1	1	1	1	1	**8**
Peloso et al. [28]	1	1	1	0	0	1	1	1	1	**7**
Peng et al. [29]	1	1	1	0	1	0	1	1	1	**7**
Mamalaki et al. [30]	1	1	1	1	0	1	1	0	1	**7**
Sun et al. [43]	1	1	1	0	1	0	1	0	1	**6**
Clark et al. [22]	1	1	1	0	1	0	1	1	1	**7**
D' Aoust et al. [23]	1	1	1	1	1	0	1	1	1	**8**
**CROSS-SECTIONAL**	**Sample Size**	**Representativeness**	**Exposure**	**Missing Data**	**Controls for AD**	**Controls for GRS**	**Outcome Assessment**	**Outcome Assessment**	**Statistical Method**	**Total Score [10]**
Mukadam et al. [17]	1	1	1	0	1	1	1	1	1	**8**
Tesi et al.[20]	1	1	1	0	1	0	1	1	1	**7**
Li et al., [31]	1	0	1	0	0	1	1	1	1	**6**
Andrews et al., [32]	1	1	1	0	1	0	1	1	1	**7**
Brenowitz et al. [33]	1	0	1	0	1	0	1	1	1	**6**
Ramanan et al., [34]	0	1	1	0	0	1	1	1	1	**6**
Saadman et al. [35]	1	1	1	0	0	1	1	1	1	**7**
Buto et al. [36]	1	1	1	0	1	0	1	1	1	**7**
Schork et al. [42]	1	1	1	0	1	1	1	1	1	**8**
Caspers et al. [44]	0	1	1	0	0	1	1	1	1	**6**
Deming et al. [46]	1	1	1	0	1	1	1	1	1	**8**

Table S3. Q-Genie assessment for genetic studies

**STUDY**	**Study Rationale**	**Outcome Selection**	**Comparison Groups Selection**	**Exposure's Technical Classification**	**Exposure's Non-technical Classification**	**Other Bias Sources**	**Sample Size & Power**	**Analyses Planning**	**Statistical Methods**	**Assumptions' Testing**	**Conclusions**	**Total Score**

Zhang et al. [47]	4	5	5	5	5	4	5	5	5	4	4	**51**
Xu et al. [21]	5	5	N/A	5	4	4	5	5	5	4	5	**47**
Tin et al. [27]	5	5	N/A	6	5	3	6	5	5	4	4	**48**
Peloso et al. [28]	5	4	N/A	5	5	4	5	5	5	4	4	**46**
Peng et al. [29]	6	4	N/A	5	5	4	7	5	5	6	5	**52**
Mamalaki et al. [30]	6	5	N/A	5	5	4	4	5	5	5	5	**49**
Sun et al. [43]	4	5	N/A	5	5	4	4	5	5	5	4	**46**
Clark et al. [22]	5	5	5	5	5	5	6	5	6	5	5	**57**
D' Aoust et al. [23]	6	5	N/A	5	5	4	5	5	5	5	5	**50**
Mukadam et al. [17]	6	4	N/A	6	5	4	7	6	5	6	5	**54**
Tesi et al.[20]	5	4	5	5	5	4	5	5	6	5	4	**53**
Li et al., [31]	5	4	N/A	5	4	4	6	4	5	4	5	**46**
Andrews et al., [32]	5	4	N/A	5	5	4	5	5	5	5	4	**47**
Brenowitz et al. [33]	5	4	N/A	4	4	4	5	5	5	5	5	**46**
Ramanan et al., [34]	5	5	N/A	5	5	5	4	5	6	5	6	**51**
Saadman et al. [35]	4	5	5	4	5	4	5	5	5	5	4	**51**
Buto et al. [36]	5	5	N/A	5	5	5	7	5	5	6	5	**53**
Schork et al. [42]	5	5	N/A	5	4	5	5	5	6	5	5	**50**
Caspers et al. [44]	5	5	N/A	5	5	5	4	5	5	5	4	**48**
Deming et al. [46]	3	5	N/A	5	4	4	5	5	5	5	5	**46**

Table S4. PRISMA 2020 Systematic Review Checklist

Section and Topic	Item #	Checklist item	Location where item is reported
**TITLE**			
Title	1	Identify the report as a systematic review.	1
**ABSTRACT**			
Abstract	2	See the PRISMA 2020 for Abstracts checklist.	Suppl. (5)
**INTRODUCTION**			
Rationale	3	Describe the rationale for the review in the context of existing knowledge.	2, 3
Objectives	4	Provide an explicit statement of the objective(s) or question(s) the review addresses.	3
**METHODS**			
Eligibility criteria	5	Specify the inclusion and exclusion criteria for the review and how studies were grouped	3, Suppl.

Section and Topic	Item #	Checklist item	Location where item is reported
		for the syntheses.	(1, 2)
Information sources	6	Specify all databases, registers, websites, organisations, reference lists and other sources searched or consulted to identify studies. Specify the date when each source was last searched or consulted.	3, 4, Suppl. (1, 2)
Search strategy	7	Present the full search strategies for all databases, registers, and websites, including any filters and limits used.	3, 4 Suppl. (1)
Selection process	8	Specify the methods used to decide whether a study met the inclusion criteria of the review, including how many reviewers screened each record and each report retrieved, whether they worked independently, and if applicable, details of automation tools used in the process.	3, 4, Suppl. (1)
Data collection process	9	Specify the methods used to collect data from reports, including how many reviewers collected data from each report, whether they worked independently, any processes for obtaining or confirming data from study investigators, and if applicable, details of automation tools used in the process.	3, 4, Suppl. (1)
Data items	10a	List and define all outcomes for which data were sought. Specify whether all results that were compatible with each outcome domain in each study were sought (e.g. for all measures, time points, analyses), and if not, the methods used to decide which results to collect.	Suppl. (1)
	10b	List and define all other variables for which data were sought (e.g. participant and intervention characteristics, funding sources). Describe any assumptions made about any missing or unclear information.	Suppl. (3)
Study risk of bias assessment	11	Specify the methods used to assess risk of bias in the included studies, including details of the tool(s) used, how many reviewers assessed each study and whether they worked independently, and if applicable, details of automation tools used in the process.	3, Suppl. (4,5)
Effect measures	12	Specify for each outcome the effect measure(s) (e.g. risk ratio, mean difference) used in the synthesis or presentation of results.	-
Synthesis methods	13a	Describe the processes used to decide which studies were eligible for each synthesis (e.g. tabulating the study intervention characteristics and comparing against the planned groups for each synthesis (item #5)).	-
	13b	Describe any methods required to prepare the data for presentation or synthesis, such as handling of missing summary statistics, or data conversions.	-
	13c	Describe any methods used to tabulate or visually display results of individual studies and syntheses.	-
	13d	Describe any methods used to synthesize results and provide a rationale for the choice(s). If meta-analysis was performed, describe the model(s), method(s) to identify the presence and extent of statistical heterogeneity, and software package(s) used.	-
	13e	Describe any methods used to explore possible causes of heterogeneity among study results (e.g. subgroup analysis, meta-regression).	-
	13f	Describe any sensitivity analyses conducted to assess robustness of the synthesized results.	-
Reporting bias assessment	14	Describe any methods used to assess risk of bias due to missing results in a synthesis (arising from reporting biases).	-
Certainty assessment	15	Describe any methods used to assess certainty (or confidence) in the body of evidence for an outcome.	-
**RESULTS**			
Study selection	16a	Describe the results of the search and selection process, from the number of records identified in the search to the number of studies included in the review, ideally using a flow diagram.	3, 4
	16b	Cite studies that might appear to meet the inclusion criteria, but which were excluded, and explain why they were excluded.	-
Study characteristics	17	Cite each included study and present its characteristics.	4 - 9
Risk of bias in	18	Present assessments of risk of bias for each included study.	Suppl.

Section and Topic	Item #	Checklist item	Location where item is reported
studies			(4, 5)
Results of individual studies	19	For all outcomes, present, for each study: (a) summary statistics for each group (where appropriate) and (b) an effect estimate and its precision (e.g. confidence/credible interval), ideally using structured tables or plots.	4 - 9
Results of syntheses	20a	For each synthesis, briefly summarise the characteristics and risk of bias among contributing studies.	-
	20b	Present results of all statistical syntheses conducted. If meta-analysis was done, present for each the summary estimate and its precision (e.g. confidence/credible interval) and measures of statistical heterogeneity. If comparing groups, describe the direction of the effect.	-
	20c	Present results of all investigations of possible causes of heterogeneity among study results.	-
	20d	Present results of all sensitivity analyses conducted to assess the robustness of the synthesized results.	-
Reporting biases	21	Present assessments of risk of bias due to missing results (arising from reporting biases) for each synthesis assessed.	-
Certainty of evidence	22	Present assessments of certainty (or confidence) in the body of evidence for each outcome assessed.	-
**DISCUSSION**			
Discussion	23a	Provide a general interpretation of the results in the context of other evidence.	9 - 11
	23b	Discuss any limitations of the evidence included in the review.	11-12
	23c	Discuss any limitations of the review processes used.	-
	23d	Discuss implications of the results for practice, policy, and future research.	12
**OTHER INFORMATION**			
Registration and protocol	24a	Provide registration information for the review, including register name and registration number, or state that the review was not registered.	3
	24b	Indicate where the review protocol can be accessed, or state that a protocol was not prepared.	3
	24c	Describe and explain any amendments to information provided at registration or in the protocol.	3
Support	25	Describe sources of financial or non-financial support for the review, and the role of the funders or sponsors in the review.	12
Competing interests	26	Declare any competing interests of review authors.	12
Availability of data, code, and other materials	27	Report which of the following are publicly available and where they can be found: template data collection forms; data extracted from included studies; data used for all analyses; analytic code; any other materials used in the review.	Suppl. (1)

Table S5. PRISMA 2020 checklist for abstracts

Section and Topic	Item #	Checklist item	Reported (Yes/No)
**TITLE**			
Title	1	Identify the report as a systematic review.	Yes
**BACKGROUND**			
Objectives	2	Provide an explicit statement of the main objective(s) or question(s) the review addresses.	Yes
**METHODS**			
Eligibility criteria	3	Specify the inclusion and exclusion criteria for the review.	No

Section and Topic	Item #	Checklist item	Reported (Yes/No)
Information sources	4	Specify the information sources (e.g. databases, registers) used to identify studies and the date when each was last searched.	Yes
Risk of bias	5	Specify the methods used to assess risk of bias in the included studies.	No
Synthesis of results	6	Specify the methods used to present and synthesize results.	No
**RESULTS**			
Included studies	7	Give the total number of included studies and participants and summarise relevant characteristics of studies.	Yes
Synthesis of results	8	Present results for main outcomes, preferably indicating the number of included studies and participants for each. If meta-analysis was done, report the summary estimate and confidence/credible interval. If comparing groups, indicate the direction of the effect (i.e. which group is favoured).	Yes
**DISCUSSION**			
Limitations of evidence	9	Provide a brief summary of the limitations of the evidence included in the review (e.g. study risk of bias, inconsistency, and imprecision).	No
Interpretation	10	Provide a general interpretation of the results and important implications.	Yes
**OTHER**			
Funding	11	Specify the primary source of funding for the review.	No
Registration	12	Provide the register name and registration number.	No

From: Page MJ, McKenzie JE, Bossuyt PM, Boutron I, Hoffmann TC, Mulrow CD, et al. The PRISMA 2020 statement: an updated guideline for reporting systematic reviews. BMJ 2021;372:n71. doi: 10.1136/bmj.n71
